# Acanthosis Nigricans in Children and Adolescents with Type 1 Diabetes or Obesity: The Potential Interplay Role between Insulin Resistance and Excess Weight

**DOI:** 10.3390/children8080710

**Published:** 2021-08-18

**Authors:** Valeria Calcaterra, Annalisa De Silvestri, Laura Schneider, Miriam Acunzo, Viola Vittoni, Giulia Meraviglia, Francesco Bergamaschi, Gianvincenzo Zuccotti, Chiara Mameli

**Affiliations:** 1 Pediatric and Adolescent Unit, Department of Internal Medicine, University of Pavia, 27100 Pavia, Italy; 2 Department of Pediatrics, Children’s Hospital “Vittore Buzzi”, 20154 Milan, Italy; laura.schneider@asst-fbf-sacco.it (L.S.); miriam.acunzo@unimi.it (M.A.); giulia.meraviglia@unimi.it (G.M.); francesco.bergamaschi@unimi.it (F.B.); gianvincenzo.zuccotti@unimi.it (G.Z.); chiara.mameli@unimi.it (C.M.); 3 Biometry & Clinical Epidemiology, Scientific Direction, Fondazione IRCCS Policlinico San Matteo, 27100 Pavia, Italy; a.desilvestri@smatteo.pv.it; 4 Department of Biomedical and Clinical Science, “Luigi Sacco”, University of Milano, 20142 Milano, Italy; 5 Pediatric Unit, Fondazione IRCCS Policlinico San Matteo and University of Pavia, 27100 Pavia, Italy; viola.vittoni@gmail.com

**Keywords:** type 1 diabetes, obesity, acanthosis nigricans, insulin resistance, children, adolescents

## Abstract

Acanthosis nigricans (AN) is associated with obesity and type 2 diabetes, where insulin resistance (IR) is considered a predisposing factor. IR can also affect patients with type 1 diabetes (T1D). We evaluated the prevalence of AN in patients with T1D compared to subjects with obesity in order to define the interplay between IR and excess weight. We considered 138 pediatric patients who presented with T1D and 162 with obesity. As controls, 100 healthy normal-weight subjects were included. A physical examination with the detection of AN and biochemical assessments was performed. IR was calculated by using the homeostasis model assessment for IR in patients with obesity and the estimated glucose disposal rate in T1D. The AN prevalence was higher in T1D and obese subjects compared with controls in whom AN was not detected (*p* = 0.02 and *p* < 0.001, respectively). A greater number of AN cases were observed in subjects with obesity compared with T1D (*p* < 0.001). Patients with AN were older than subjects without AN (*p* = 0.005), and they had higher body mass index (BMI) values, waist circumference (WC), fasting triglycerides and blood pressure (all *p* < 0.001). Thirty-five patients with AN exhibited IR with an association between AN presence and IR in patients with obesity (*p* < 0.001). In T1D, there was an association between AN and being overweight/obese (*p* = 0.02), independently of IR. AN is a dermatological condition associated with obesity. In T1D, the presence of AN was significantly associated with overweight status or obesity but not IR. The presence of AN in the absence of IR supports the interplay role between impaired insulin signaling, IR and excess weight in the pathogenic mechanism.

## 1. Introduction

Several metabolic diseases are associated with skin manifestations that may precede, succeed or be concomitant with the onset of the pathological condition [1,2]. Regarding the scope of this study, tissue resistance to insulin is a major feature underlying the acanthosis nigricans (AN) development [3,4].

AN is a skin manifestation characterized by silky, hyperpigmented and hyperkeratotic plaques with ill-defined borders that typically appear in intertriginous areas [5,6]. The pathogenesis of AN is not fully understood [5,6]. However, in the pediatric population, AN is more commonly related with obesity and type 2 diabetes [5]. In this regard, a hyperinsulinemic state due to peripheral insulin resistance (IR) is considered a predisposing factor. Elevated serum insulin levels cause a direct or indirect interaction between insulin and insulin-like growth factor-1 receptors (IGF-R) leading to the proliferation of keratinocytes and fibroblasts in the peripheral tissues (at skin level) [6].

IR is defined as the inability of a known quantity of exogenous or endogenous insulin to increase glucose uptake and utilization [4]. IR is the main pathogenic mechanism underlying type 2 diabetes, hypertension, cardiovascular disease and obesity. However, IR can also affect insulin deficient patients with type 1 diabetes (T1D) and may be associated with metabolic, macrovascular and microvascular complications [7,8,9]. Even though patients with T1D usually present normal body weights, the prevalence of overweight status and obesity in individuals with T1D is increasing in parallel to the global population trends in weight gain [10,11].

Data on the prevalence of AN in T1D are described in adults [11,12,13,14], and a limited number of studies are also reported in pediatric age [14,15,16].

The aim of this study was to consider the prevalence of AN in children and adolescents affected by T1D compared with subjects with obesity in order to explore the potential association between AN and impaired insulin signaling and excess weight in two different conditions of insulin secretion, such as insulin deficiency in T1D in contrast to hyperinsulinemia in obesity.

## 2. Patients and Methods

### 2.1. Patients

We retrospectively considered 138 children and adolescents diagnosed with T1D (aged 12.5 ± 4.5 years; mean duration of diabetes 5.64 ± 4.84 years) and 162 with obesity (12.1 ± 4.1 years). Patients were enrolled between September 2020 and May 2021 at the outpatients’ clinic of Children’s Hospital Vittore Buzzi, Milano. All patients with T1D were receiving insulin therapy and no other medications. Exclusion criteria included the following: obese patients with known secondary obesity conditions using any medications other than insulin therapy and concomitant chronic or acute illnesses. All patients underwent an auxological evaluation and biochemical assessments. The presence of AN over the neck and axilla was recorded [17]. As a control group, 100 age-matched healthy normal weight subjects were included.

### 2.2. Anthropometric Measurements

Height, weight, calculated body mass index (BMI), waist circumference (WC), pubertal stage and blood pressure (BP) were measured.

Weight, height, WC and blood pressure were measured as previously described [18]. BMI was calculated by dividing the patient’s weight in kilograms by the square of the height in meters and then transformed into BMI z scores by using the World Health Organization (WHO, Geneva, Switzerland) reference values [19]. The condition of obesity was defined as a BMI-for-age value over +2 and standard deviation (SD) and overweight as a BMI-for-age value over +1 SD.

The stages of puberty were classified according to Marshall and Tanner [20,21] as the following: prepuberty, boys with genital Tanner 1 and girls with breast Tanner 1; early puberty, boys with genital Tanner 2–3 and girls with breast Tanner 2–3; late/post puberty, boys with genital Tanner ≥ 4 and girls with breast Tanner ≥ 4.

Systolic (SBP) and diastolic (DBP) blood pressures were measured twice using a mercury sphygmomanometer after the participant sat comfortably for 5 min, with an appropriately sized cuff on the right arm, which was slightly flexed at the heart level. The second BP measurement was used for analysis.

### 2.3. Metabolic Profile

Blood samples were performed the morning after an overnight fast. Fasting blood glucose (FBG), insulin, total cholesterol, high density lypoprotein (HDL)-cholesterol and trygliceride (TG) were measured by clinical chemistry methods with an Advia XPT instrument (Siemens Healthcare, Erlangen, Germany) as previously described [22,23].

### 2.4. Insulin Resistance Measurement

IR in patients with obesity was calculated by using the homeostasis assessment model for insulin resistance (HOMA-IR), according to the following formula: insulin resistance  =  (fasting plasma insulin in mUI/L ×  fasting plasma glucose in mmol/L)/22.5. Impaired insulin sensitivity (ISI) was defined as a HOMA-IR ≥ 97.5th percentile for age and sex [24].

IR in patients with T1D was determined with the estimated glucose disposal rate (eGDR) and is calculated as follows: eGDR (mg = kg = min) = 21.158 +(−0.09 * waist circumference) + (−3.407 * HTN) + (−0.551 * HbA1c) where HTN is hypertension (0 = no; 1 = yes), defined with systolic (SBP) or diastolic (DBP) blood pressures values exceeding the 95th percentile for age and sex [25]. Importantly, eGDR shows a good correlation with IR when measured with an euglycemic-hyperinsulinemic clamp and has been validated for the estimation of insulin sensitivity in T1D subjects [26]. For these reasons, we utilized eGDR as an indicator of IR. According to Chillaron et al., we used an eGDR level less than eGDR of 8.77 mg kg^−1^ min^−1^ as a cutoff for IR [26].

### 2.5. Metabolic Syndrome Definition

Metabolic syndrome (MS) was defined as the presence of at least 3 of the following components [27]:

(1) Waist circumference was ≥ 90th percentile;
(2) Serum triglyceride levels 150 mg/dl or treatment for hypertriglyceridemia;
(3) Serum HDL cholesterol levels < 40 mg/dL or on drug treatment;
(4) Blood systolic pressure ≥ 130 mmHg and/or blood diastolic pressure ≥ 85 mmHg or on anti-hypertensive medication;
(5) Fasting plasma glucose ≥ 100 mg/dL and/or HOMA-IR > 97.5th percentile in patients with obesity and controls. As markers of glucometabolic derangement, we also used it because it precedes glucose abnormalities by playing a crucial role in the pathogenesis of impaired glucose tolerance transition [28].

As all T1D patients were on insulin by definition, the fasting plasma glucose criteria were taken as positive for all subjects. 

## 3. Statistical Analysis

Stata 16 (StataCorp, College Station, TX, USA) was used to perform all analyses. Data were described as the mean, standard deviation (SD), median and 25th–75th percentiles if continuous and as counts and percentages if categorical. The Shapiro–Wilks test was used to determine the normality of data. Comparisons of quantitative variables between groups were made with the t-test for independent data or one-way ANOVA followed by 2 × 2 post-hoc comparisons with Scheffè correction. The association of categorical variables was assessed with the Fisher’s exact test. For the purpose of this analysis, association between AN and biomarkers was performed separately in type 1 diabetes and obesity groups. Logistic regression models were fitted with AN as dependent variable; non-collinear parameters significantly different between patients with or without acanthosis in T1D or obesity group were the independent factors. The results are reported as odds ratio (OR) with 95% confidence interval (95% CI). All tests were 2-sided. A *p*-value < 0.05 was considered statistically significant.

## 4. Results

In Table 1, the clinical and biochemical data of the enrolled patients and controls are reported.

### 4.1. Distribution of Acantosis Nigricans

AN was detected in 63/400 (15.75%) of the enrolled subjects. The prevalence of AN was higher in patients with T1D (7/138, 5.07%) and obesity (56/162, 34.57%), compared with controls in whom AN was not detected (*p* = 0.02 and *p* < 0.001, respectively). A greater number of AN cases in subjects with obesity compared with T1D were also observed (*p* < 0.001). No sex bias with AN cases was noted while patients with AN were older than subjects without AN (*p* = 0.005) (see Table 2).

### 4.2. Acanthosis Nigricans and Clinical and Biochemical Parameters

As reported in Table 2, a difference in clinical and metabolic parameters was noted in subjects with or without AN. Patients with AN had higher BMI values (*p* < 0001 and *p* = 0.047), WC (*p* < 0.001 in obesity group), fasting TG (*p* < 0.001 in obesity group), SBP (*p* < 0.001 in obesity group) and DBP (*p* = 0.025 and 0.020), fasting insulin and HOMA (*p* < 0.001 in obesity group) and estimated glucose disposal rate (*p* = 0.033 in type 1 diabetes).

Thirty-five of the patients with AN (35/63; 55.5%) exhibited IR (34 with obesity and 1 with T1D) with a significant association between the presence of AN and IR in patients with obesity (*p* < 0.001). In T1D, the presence of AN was significantly associated with overweight status or obesity (*p* = 0.02) but not IR. An association between AN and puberty was noted (*p =* 0.03).

Metabolic syndrome was detected in 16/162 (9.9%) subjects with obesity, in 22/138 (15.9%) patients with T1D and in 1/100 (1%) controls (*p* < 0.001) with significant difference in patients with or without AN (*p* = 0.01).

In Table 3, logistic analysis to consider the main potential confounding factors for AN is reported. Overweight and obesity in subjects with T1D and BMI and triglycerides in the obesity group are independent risk factors.

## 5. Discussion

AN is described as a velvety darkening of the skin that usually involves intertriginous areas. AN prevalence varies among different age groups and ethnicities, reaching 25% in the general population and ranges from 49.2% to 58.2% in children or adolescents with obesity [29,30]. AN has been previously described in children with obesity [31,32,33] and less frequently in pediatric patients with T1D [14,15,16]. In our pediatric population, we described an overall AN prevalence of 15.75%, with a higher prevalence in patients with T1D (5%) and obesity (34.57%) compared with controls. No sex bias was noted. In accordance with the literature, AN was more common in our population in adolescents than in younger children, which can be explained by the longer duration of being obese and/or having IR past puberty [30].

A relationship between AN and IR has also been described [3,4]. Although IR is generally associated with obesity and type 2 diabetes, it has also been described in T1D [8,9], particularly during puberty and inter-current illness. Some youths with T1D and IR do not have the typical central adiposity phenotype [8], and IR is more likely related to glucotoxicity, which damages glucose transport systems and post-insulin receptor action pathways [34].

The role of metabolic derangement is described [14,15,16,17,31,32,33]. In our study, the presence of AN in subjects with obesity was associated with IR, while AN seemed to be related more with excess weight than IR in the T1D group. Although the exact pathogenic mechanisms of AN are not fully understood, these data support an interplay between impaired insulin signaling and increased insulin resistance, as well as adipose tissue (AT) dysfunction. Moreover, other components of MS [14,15,16,17,31,32,33] and an elevated chronic inflammation status should be also considered [30].

The activation of IGF receptors by hyperinsulinemia, triggering dermal fibroblast and epidermal keratinocyte proliferation has been proposed as a pathogenic mechanism. Hyperinsulinemia in AN patients may increase the binding of insulin and IGF-R and may also reduce IGF-binding proteins (IGFBPs), thus increasing biologically active IGF-1; this results in the development of hyperkeratosis and papillomatosis [6,35,36,37]. The presence of AN in patients with ponderal excess without hyperinsulinemia and/or IR as in our T1D group supports the AT dysfunction, and an impaired adipocyte condition might represent a novel link between obesity and AN [30].

In fact, obesity is characterized by an increase in AT (hypertrophy and hyperplasia), which induces hypoxia, apoptosis and cell stress, leading to an altered AT environment and alteration in tissue homeostasis. The dysregulated secretion by adipocytokines, such as leptin and adiponectin, as well as increased secretion of proinflammatory cytokines, such as interferon-γ, interleukin 6 and tumor necrosis factor-α, results in an infiltration of inflammatory immune cells in the AT [38]. The chronic inflammation may not only modulate the metabolic profile but could also contribute to the development of skin diseases [39], including AN [30], which is a skin manifestation in global inflammatory-related diseases.

By highlighting the differences in lipid and blood pressure levels and the significant correlation between AN and MS, in our population the importance of the dismetabolism in patients with AN has also been confirmed, thus supporting the identification of AN as a high-risk sign of metabolic abnormalities [30]. The role of AN as a precursor to IR and/or obesity should also be considered [17,30]. Skin phototypes, genetic makeup and epigenetic factors should also be considered in the pathogenesis of AN [17].

We are aware of the limitations of this study that are mainly related to the use of indirect IR measurements. The hyperinsulinemic-euglycemic glucose clamp is the gold standard method for assessing insulin sensitivity, however, it is invasive and not practical in pediatrics. Secondly, even though AN can affect any part of the skin, including the neck, axillae, groin, anogenital areas, face, eyelids, supra-alar nasal creases, inner thigh, antecubital fossa, popliteal fossa, umbilicus, palms, soles, knuckles, nipples and areolas, we only considered the neck and axillae in our study because these are the most common sites and are more accessible than others for screening [17]. Additionally in T1D, the influence of repeated insulin injections in the induction of AN was not considered [39]. Finally, no data on family history of type 2 diabetes and/or other dismetabolisms are recorded. Despite these limitations, our preliminary study indicates that AN could be used as a marker of IR as well as a clinical sign of chronic AT inflammation.

## 6. Conclusions

AN is a dermatological condition associated with obesity. In T1D, the presence of AN was significantly associated with overweight status or obesity but not IR. The presence of AN in the absence of IR supports the interplay role between impaired insulin signaling, IR and excess weight in the pathogenic mechanism. AN screening may help to identify coexisting comorbidities related to adiposity or patients at high risk.

## Figures and Tables

**Table 1:** Clinical and biochemical features of the patients and controls.

Features	Controls (*n* = 100)	Type 1 Diabetes (*n* = 138)	Obesity (*n* = 162)	*p* ^
Age (years)	12.1 ± 2.6	12.5 ± 4.5	12.1 ± 4.1	ns
Sex (M/F)	61/49	65/73	86/76	ns
Body mass index (Kg/m^2^)	17.6 ± 2.7	20.7 ± 4.3	27.9 ± 5.3	<0.001 a b c
Waist circumference (cm)	63.3 ± 8.6	69.9 ± 13.7	87.3 ± 12.6	<0.001 a b c
HbA1c (%)	4.9 ± 0.4	7.9 ± 1.3	4.9 ± 1.2	<0.001 a c
Fasting blood glucose (mg/dL)	74.6 ± 7.7	124.5 ± 46.1	79.4 ± 11.5	<0.001 a c
Fasting insulin (IU/mL)	6.3 ± 4.1	np	14.3 ± 4.2	<0.001
HOMA-IR (%)	1.2 ± 0.8	np	2.8 ± 2.5	<0.001
Estimated glucose disposal rate (mg kg^−1^ min^−1^)	np	10.5 ± 1.5	np	np
Fasting triglycerides (mg/dL)	54.3 ± 22.3	70.0 ± 40.9	67.2 ± 33.8	0.003 a b
Total cholesterol (mg/dL)	156.4 ± 28.4	166.6 ± 33.5	157.7 ± 29.2	0.017 a c
HDL cholesterol (mg/dL)	54.7 ± 10.8	57.7 ± 12.0	65.3 ± 35.5	0.003 b c
Systolic blood pressure (mmHg)	100.7 ± 9.9	108.8 ± 12.7	110.8 ± 15.0	<0.001 a b
Diastolic blood pressure (mmHg)	63.1 ± 8.7	68.0 ± 8.1	67.6 ± 10.1	<0.001 a b

np: not performed ^ oneway ANOVA followed by 2 × 2 post-hoc comparisons with Scheffè corrections; a: *p* < 0.05 controls vs. type I diabetes; b: *p* < 0.05 controls vs. obesity; c: *p* < 0.05 obesity vs. type I diabetes; HbA1c: glycated haemoglobin; HOMA-IR: homeostasis assessment model for insulin resistance; HDL: High-density lipoprotein.

**Table 2:** Clinical and biochemical features of the patients with or without acanthosis nigricans.

Parameters	Acanthosis Nigricans						*p* * Obesity	*p* * Type 1 Diabetes
	Yes N = 63 (Type 1 Diabetes *n* = 7; Obesity *n* = 56)			No N = 337 (Type 1 Diabetes *n* = 131; Obesity *n* = 106)				
	Total	Type 1 Diabetes	Obesity	Total	Type 1 Diabetes	Obesity		
Age (years)	13.77 ± 2.30	14.49 ± 3.77	13.69 ± 2.09	12.19 ± 4.41	12.50 ± 5.61	11.60 ± 3.66	<0.001	ns
Body mass Index (kg/m^2^)	31.62 ± 5.51	23.86 ± 4.95	32.60 ± 4.84	25.94 ± 3.92	20.56 ± 4.21	25.50 ± 3.74	<0.001	0.047
Waist circumference (cm)	94.34 ± 14.59	72.79 ± 18.92	97.25 ± 11.31	71.74 ± 13.50	69.80 ± 13.53	82.3 ± 9.99	<0.001	ns
Pubertal stage							0.033	ns
Early	5	2	3	59	44	15		
Middle	11	3	8	55	28	27		
late	47	2	45	123	59	64		
HbA1c (%)	np	8.81 ± 1.64	5.05 ± 0.38	np	7.85 ± 1.31	4.91 ± 0.28	ns	ns
Fasting blood glucose	np	1180 ± 28.3	81.09 ± 13.92	np	122.0 ± 35.1	79.60 ± 10.01	ns	ns
Fasting insulin	np	np	21.25 ± 12.83	np	np	10.58 ± 6.64	<0.001	np
HOMA-IR	np	np	4.32 ± 3.19	np	np	2.02 ± 1.54	<0.001	np
Estimated glucose disposal rate (mg kg^−1^ min^−1^)	np	9.26 ± 2.01	np	np	10.55 ± 1.52	np	np	0.033
Fasting triglycerides (mg/dL)	82.60 ± 38.85	83.71 ± 21.58	82.46 ± 40.70	61.19 ± 32.88	69.30 ± 41.66	57.10 ± 25.45	<0.001	ns
Total cholesterol (mg/dL)	160.48 ± 31.53	165.64 ± 33.63	158.36 ± 26.60	161.46 ± 27.72	185.00 ± 26.39	157.4 ± 30.77	ns	ns
HDL cholesterol (mg/dL)	60.55 ± 29.63	59.57 ± 21.82	60.74 ± 31.10	59.04 ± 21.04	57.57 ± 11,41	68.03 ± 37.81	ns	ns
Systolic blood pressure (mmHg)	116.77 ± 11.93	112.86 ± 12.54	117.27 ± 11.89	105.68 ± 13.28	108.53 ± 12.70	107.27 ± 15.40	<0.001	ns
Diastolic blood pressure (mmHg)	70.60 ± 7.70	75.00 ± 5.00	70.05 ± 7.84	65.78 ± 9.47	67.63 ± 8.13	66.29 ± 11.01	0.025	0.020

* *t*-test for independent data; np: not performed, ns: not significant; HbA1c: glycated haemoglobin; HOMA-IR: homeostasis assessment model for insulin resistance; HDL: High-density lipoprotein.

**Table 3:** Logistic analysis of the main potential confounding factors for AN is reported.

Parameters	Odds Ratio	*p*	95% Conf. Interval
	Type 1 Diabetes		
Overweight/obesity *	4.26	0.04	1.66–10.9
Estimated glucose disposal rate	0.73	0.37	0.37–1.45
Diastolic blood pressure	1.14	0.17	0.94–1.38
	**Obesity**		
Body Mass Index	2.00	<0.001	1.49–2.69
HOMA-IR	1.12	0.33	0.88–1.42
Age	1.01	0.93	0.77–1.31
Triglycerides	1.02	0.007	1.00–1.03
Systolic blood pressure	1.00	0.73	0.97–1.04

HOMA-IR: homeostasis assessment model for insulin resistance. * as categorical variable.

## Data Availability

The data presented in this study are available upon request from the corresponding author.
